# Erosion characteristics of different reclaimed substrates on iron tailings slopes under simulated rainfall

**DOI:** 10.1038/s41598-020-61121-z

**Published:** 2020-03-06

**Authors:** Chunjuan Lv, Rutian Bi, Xingxing Guo, Dan Chen, Yansong Guo, Zhanjun Xu

**Affiliations:** College of Resources and Environment, National Experimental Teaching Demonstration Center, Shanxi Agricultural University, Taigu, Shanxi 030801 China

**Keywords:** Restoration ecology, Restoration ecology

## Abstract

Water-induced erosion of iron tailings is a serious problem affecting ecological restoration, but, little is known about how the occurrence of erosion on tailings slopes and types of reclaimed substrates that are beneficial to reducing slope erosion. This study measured the slope erosion characteristics of six reclaimed substrates including loose tailings (LT), crusty tailings (CT), tailings incorporating mushroom residues (TM), tailings incorporating soil (TS), tailings incorporating soil and mushroom residues (TSM) and soil (S) in experimental soil flumes under three simulated intermittent rainfall events, with intensity of 60, 90 and 120 mm h^−1^ for the first, second and third event, respectively. Significant differences (*p* < *0.05*) were found in erosion characteristics among the six reclaimed substrates. TM had the lowest sediment yield but the highest runoff volume without obvious rills. LT, CT and TS had the highest sediment yield rates and severe slope erosion morphology. With the increased number of rainfall events, the runoff rates of the six substrates all increased, but only the sediment yield rates of LT, CT and TS increased, the sediment yield rates of other substrates increased first and then decreased. Therefore, adding agricultural organic wastes such as mushroom residues to tailings and reducing soil addition may be an effective way to reduce erosion and promote ecological restoration in soilless tailings areas.

## Introduction

Mining is essential for maintaining society’s current life style and economic development. However, mining also impacts the environment^1–3^. One of the most significant environmental impacts from mining is soil erosion, which has received increasing attention^3–8^. Waste dumps generated from mining usually consist of unconsolidated waste rock, parent material, tailings or their mixtures^9,10^. The waste dumps are piled on the land surface in the form of overburden dump. They usually become the material source for debris flow, river sediment, and hyper-concentrated sediment flow of artificially accelerated erosion^11,12^ as well as the source of wind erosion^13,14^.

The erosion forms and characteristics of mining waste dumps are different from those occuring naturally^5^. Accumulated overburden dump material is prone to gravity erosion^12^, causing abrupt increases or decreases in sediment yield^15^. Erosion in coal waste slag has shown extreme sediment fluctuation at the beginning of runoff, accompanied by local debris flow^16^. Martín Duque *et al*.^1^ observed that tailing deposits underwent severe erosion after long-term abandonment, and as a result, caused a mosaic-like erosion and sedimentary landforms with gully formations upon and piping within deposits. Therefore, at a larger scale, it is important to reconstruct stable post-mining landforms and control erosion, which have been claimed in mine reclamation regulations in different countries^17,18^. Most of the traditional landform reconstruction is in the form of terraces and contoured banks^3^. Recent studies indicated that geomorphic-based mining reclamation with stable slopes and drainage networks at a watershed scale can reestablish an approximate steady-state or dynamic equilibrium close to the surrounding natural landform^3,19,20^.

Slope erosion is a serious problem at both watershed and dump scales, especially for long, steep slopes composed of unconsolidated erodible materials. Riley^21^ reported that the slope erodibility in mining areas was 10–100 times greater than that of the natural slope, and the erosion rate was noted to be above 100 Mg (MT) ha^−1^ yr^−1^ for blends of tailing deposits^1^. The mining waste particle size or contents and position influence runoff and infiltration^1,22–25^. The gravel content had little effect on the runoff rate of loess waste dump, but the sediment rate greatly decreased with an increase in gravel content^7,26^. Soil erosion is a particle size-selective process, especially for the non-homogeneous mining waste^27^, and fine particles and nutrients are easily removed^28^, which severely affects the sustainability of ecological restoration^29^. To improve the physical and chemical properties of degraded soil, different types of composts have been applied to soil as amendments to improve their fertility^30^ and enhance their capacity of water storage and retention or structural stability^28^. Mushroom residues, as organic compost, have also been widely used to amend soil^30–32^. Mulching, such as straw, residue cover and vegetal cover has been found to be effective in reducing soil and water losses^33^. Organic matter and clay maintain aggregate stability by binding soil particles which improves the soil structure and strengthens the resistance to erosion^34^. Runoff rate and sediment concentration have been found to be closely related to intrinsic soil properties^35,36^, especially the content of fine particles with aggregate structure^14,37,38^.

China is one of the largest mining countries in the world, and most of the iron mines are low grade ore. The amount of iron tailings accounts for nearly 1/3 of the total tailings as a result of the great amount of excavation and discard in China^5^. Tailings are characterized by very fine ore powders and heaped into 35–40° steep slopes, which are prone to cause erosion^39^. There have been many studies on erosion road embankments and mine waste dumps^9,40,41^, Campbell *et al*.^42^ evaluated the erodibility and hydrological response of 10 slope-forming materials derived from an iron ore mine in West Africa. However, tailings slopes are rarely considered with respect to water erosion.

This study was conducted to investigate how tailing erosion under three rainfall events was affected by mixing soil and mushroom residues. The objectives were to: (i) determine the water erosion characteristics and influencing factors of iron tailings, (ii) investigate the influence of substrate types and intermittent rainfall events on runoff, sediment yield, and slope micro-topograph, and (iii) examine the role of mushroom residues on tailings erosion control.

## Results

### Runoff processes

#### Time to runoff initiation

**Figure 1:**
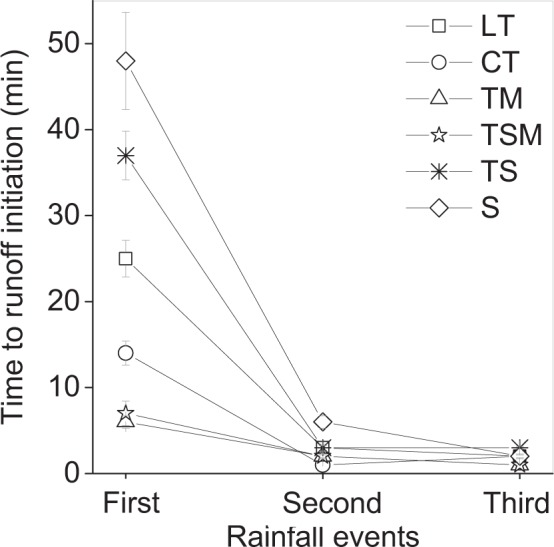
Time to runoff initiation for reclaimed substrates under three intermittent rainfall events. T, tailings; M, mushroom residues; S, soil; LT, loose tailings; CT, crusty tailings; TM, tailings incorporating mushroom residues; TS, tailings incorporating soil; TSM, tailings incorporating soil and mushroom residues. Error bars show the standard derivation among the repetitions (n = 3).

When the same slope was subjected to intermittent rainfall events, an antecedent rainfall event had a great impact on the time to runoff initiation because of the changes in surface soil properties and in slope micromorphology. The time to runoff initiation varied between 6–48 min when the first rainfall event occurred, and decreased rapidly from 1–6 min to 1–3 min during the second and third rainfall events (Fig. 1). The time to runoff initiation was also different among substrates, which decreased in the order of S > TS > LT > CT > TSM > TM. Compared with the LT and CT tailings, S and TS delayed the time to runoff initiation, and the TSM and TM accelerated the initiation of runoff.

#### Flow velocity

**Figure 2:**
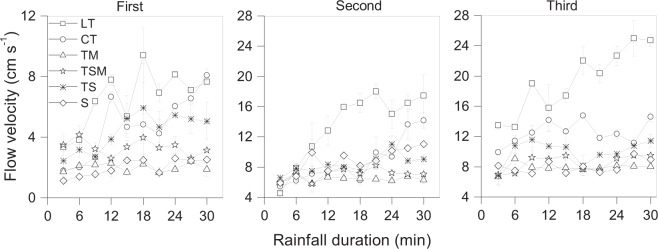
Change in flow velocity for reclaimed substrates with rainfall duration under three intermittent rainfall events. T, tailings; M, mushroom residues; S, soil; LT, loose tailings; CT, crusty tailings; TM, tailings incorporating mushroom residues; TS, tailings incorporating soil; TSM, tailings incorporating soil and mushroom residues. Error bars show the standard derivation among the repetitions (n = 3).

The mean flow velocities are shown in Fig. 2. The mean flow velocities were significantly different between the six substrates (P < 0.05). The changing trends in flow velocity could be regrouped into two types: With the increase of rainfall duration, LT, CT and TS showed upward trends with greater variability; TM, TSM and S showed horizontal fluctuations with less variability.

The flow velocities increased with increased number of rainfall events for all six substrates. When the three rainfalls events occurred in sequence, the mean flow velocities varied from 1.13–9.41, 4.56–18.01 and 6.68–25.00 cm s^−1^, respectively.

#### Runoff rate

**Figure 3:**
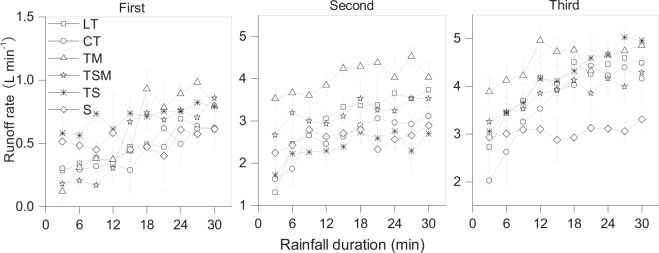
Change in runoff rate with rainfall duration under three intermittent rainfall events. T, tailings; M, mushroom residues; S, soil; LT, loose tailings; CT, crusty tailings; TM, tailings incorporating mushroom residues; TS, tailings incorporating soil; TSM, tailings incorporating soil and mushroom residues. Error bars show the standard derivation among the repetitions (n = 3).

The runoff rate of S fluctuated horizontally with increasing rainfall duration, while the runoff rate of the other substrates exhibited fluctuating uprising tendency (Fig. 3). There were significant differences in runoff rates among six substrates (P < 0.05). Runoff rate increased with increased number of rainfall events. Particularly, the runoff rate in the second rainfall event increased by 5.4 times compared to that in the first rainfall event.

The relative size of runoff rates from the six substrates differed totally under three rainfall events (Fig. 3 and Table 1). In the first rainfall event, the runoff rate of TS was high throughout the rain event. The runoff rates of TM and TSM were low in the first 15 min and rapidly increased during the subsequent rainfall process, approaching or exceeding that of TS. However, in the second and the third rainfall events, the highest runoff rate was observed from TM, followed by LT and CT; the runoff rate with S was relatively lower and varied steadily all the time, which differed from the other substrates.

**Table 1:** Cumulative runoff volume, cumulative sediment yield and sediment concentration for six reclaimed substrates under three intermittent rainfall events.

Items	Rainfall events	Substrates					
		LT	CT	TM	TSM	TS	S
Cumulative runoff volume ± SD (L)	First	14.2 ± 1.6 cd	13.1 ± 0.7d	18.1 ± 1.1b	16.9 ± 1.9bc	21.2 ± 0.6a	15.5 ± 0.9bcd
	Second	91.4 ± 5.8b	78.6 ± 2.5c	120.5 ± 1.6a	96.0 ± 2.8b	72.0 ± 2.6c	78.2 ± 1.1c
	Third	120.2 ± 3.1bc	109.8 ± 7.1d	135.9 ± 1.7a	115.4 ± 3.1 cd	126.1 ± 1.9b	92.2 ± 1.1e
	Sum	225.8	201.5	274.5	228.3	219.3	185.9
Cumulative sediment yield ± SD (g)	First	453.4 ± 12.1a	402.5 ± 42.2a	103.7 ± 4.9c	193.6 ± 13.8b	431.8 ± 22.2a	18.9 ± 3.6d
	Second	15106.2 ± 688.4a	9824.5 ± 381.0b	3334.2 ± 76.9e	6721.0 ± 195.3c	13806.7 ± 1033.8a	5009.0 ± 222.4d
	Third	21886.2 ± 721.9a	15507.1 ± 971.5b	1447.2 ± 231.0d	6053.4 ± 347.6c	21895.6 ± 939.4a	4723.2 ± 146.0c
	Sum	37445.8	25734.1	4885.1	12968.0	36134.1	9751.1
Sediment concentration ± SD (g/L)	First	31.8 ± 4.3a	30.7 ± 1.7a	5.7 ± 0.1d	11.5 ± 0.4c	20.4 ± 0.5b	1.2 ± 0.2d
	Second	165.2 ± 2.9b	124.9 ± 8.9c	27.7 ± 0.3e	70.0 ± 0.01d	191.9 ± 7.5a	64.1 ± 3.8d
	Third	182.1 ± 10.7a	141.2 ± 0.3b	10.7 ± 1.6d	52.5 ± 1.6c	173.7 ± 4.9a	51.2 ± 2.2c
	Mean	126.4	99.0	14.7	44.7	128.7	38.8

T, tailings; M, mushroom residues; S, soil, LT, loose tailings; CT, crusty tailings; TM, tailings incorporating mushroom residues; TS, tailings incorporating soil; TSM, tailings incorporating soil and mushroom residues. S.D. means the standard derivation among the repetitions (n = 3). Different letters show significant difference at *P* < 0.05 according to the LSD test for different substrates with the same rainfall event.

### Sediment process

#### Sediment yield rate

**Figure 4:**
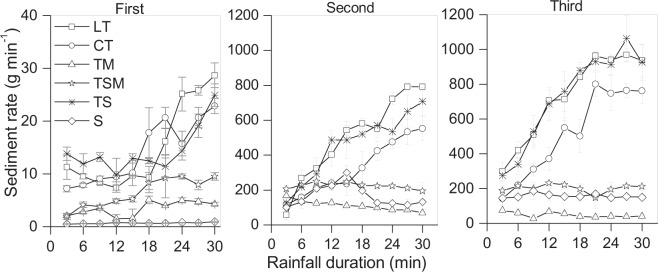
Change in sediment yield rate with rainfall duration under three intermittent rainfall events. T, tailings; M, mushroom residues; S, soil; LT, loose tailings; CT, crusty tailings; TM, tailings incorporating mushroom residues; TS, tailings incorporating soil; TSM, tailings incorporating soil and mushroom residues. Error bars show the standard derivation among the repetitions (n = 3).

Significant differences in sediment yield rate were detected among three rainfall events and six substrates (*P* < 0.05) (Fig. 4). For each rainfall event, the LT, CT and TS had higher sediment yield rates than TSM, TM and S. After the three rainfall events, the cumulative sediment yield of LT, CT and TS reached up to 37445.8, 25734.1 and 36134.1 g min^−1^, respectively. TM had the lowest cumulative sediment yield of 4885.1 g min^−1^ (Table 1).

The sediment processes in LT, CT and TS were also completely different from those in TM, TSM and S (Fig. 4). The sediment yield rates in LT, CT and TS increased rapidly with rainfall duration, and TM, TSM and S showed a slow increase or decrease with the increase of rainfall duration.

Under the three intermittent rainfall events, the cumulative sediment yield of LT, CT and TS consistently increased; but the cumulative sediment yield of TM, TSM and S first increased and then decreased (Table 1). Between the first two rainfall events, the increase of sediment yield was the most obvious, the mean sediment yield of S increased by up to 265 times, the remaining substrates increased by about 30 times.

#### Sediment concentration

The sediment concentration of LT and CT increased continuously with increasing number of rainfall events (Table 1), while that of other substrates increased in the second rainfall event and then decreased in the third rainfall event.

There was a significant difference in sediment concentrations between substrates (P < 0.05). LT, CT and TS had higher sediment concentrations than the other substrates because of the synergistic effects of high runoff volume and high sediment yield. The sediment concentration of TM was the lowest as a result of the highest runoff volume and the lowest sediment yield.

### Slope micro-topographic characteristics and erosion correlations

#### Slope micro-topographic characteristics

Mean relief amplitude (RA) varied from 2.5 to 38.9 mm. Gully area (GA) and gully density (GD) varied from 0 to 8.6% and from 0 to 12750.6 km km^−2^, respectively (Table 2). There were significant differences in the RA, GA and GD among substrates under the same rainfall event. As a whole, LT, CT and TS had greater RA, GA and GD than the other substrates. For TM, the RA and GA always equal to 0, because there was no rill in the slope surface except for slight pits induced by splash erosion. Intermittent rainfall events aggravated the change of slope morphology: in general, RA and GA increased with increased number of rainfall events, GD increased rapidly first and then decreased slightly with increased number of rainfall events.

**Table 2:** Slope micro-topographic factors for six substrates under three intermittent rainfall vents.

Rainfall events	Substrates	RA ± SD (mm)	GA ± SD (%)	GD ± SD Km/Km^2^)	f_D_ ± SD	n_m_ ± SD
First	LT	5.0 ± 0.5a	2.1 ± 0.3a	3339.4 ± 423.4a	1.9 ± 0.3b	0.032 ± 0.002c
	CT	3.3 ± 0.3b	1.1 ± 0.2b	1223.7 ± 280.9b	9.5 ± 2.9b	0.069 ± 0.010b
	TM	3.7 ± 0.3b	0 ± 0c	0 ± 0d	59.5 ± 7.3a	0.236 ± 0.019a
	TSM	5.4 ± 0.3a	0.7 ± 0.1bc	502.3 ± 87.6cd	12.8 ± 1.0b	0.098 ± 0.004b
	TS	2.5 ± 0.4c	1.1 ± 0.7b	928.6 ± 117.5bc	10.3 ± 0.4b	0.086 ± 0.001b
	S	3.0 ± 0.1bc	0 ± 0c	0 ± 0d	76.5 ± 16.5a	0.253 ± 0.029a
Second	LT	16.7 ± 1.3a	10.3 ± 1.4b	10472.1 ± 1313.1b	2.0 ± 0.5d	0.037 ± 0.005d
	CT	13.4 ± 1.2b	9.5 ± 1.1b	12750.6 ± 1652.4a	5.4 ± 0.4c	0.069 ± 0.001c
	TM	7.1 ± 0.7c	0 ± 0d	0 ± 0d	11.4 ± 0.8a	0.121 ± 0.004a
	TSM	7.8 ± 0.5c	4.7 ± 0.5c	5550.2 ± 553.3c	8.6 ± 1.5b	0.099 ± 0.007b
	TS	20.0 ± 2.6a	15.6 ± 1.2a	3667.6 ± 385.5c	3.2 ± 0.1 cd	0.056 ± 0.001c
	S	5.4 ± 0.6c	4.6 ± 0.8c	812.4 ± 134.3d	4.8 ± 2.0c	0.066 ± 0.012c
Third	LT	38.9 ± 3.1a	13.8 ± 1.1b	9551.4 ± 1160.3a	0.5 ± 0d	0.021 ± 0.001e
	CT	31.3 ± 3.0b	12.1 ± 1.5b	10635.0 ± 1225.9a	1.5 ± 0.1d	0.038 ± 0.001d
	TM	8.6 ± 0.4c	0 ± 0d	0 ± 0d	7.0 ± 1.0a	0.094 ± 0.007a
	TSM	10.6 ± 1.5c	6.3 ± 1.3c	5219.0 ± 359.4b	5.0 ± 0.2b	0.075 ± 0.001b
	TS	35.4 ± 3.3ab	28.6 ± 2.7a	3292.0 ± 408.3c	3.5 ± 0.3c	0.062 ± 0.002c
	S	6.0 ± 0.4c	7.3 ± 1.2c	1750.1 ± 175.1 cd	4.7 ± 0.7bc	0.071 ± 0.005b

RA, Relief amplitude; GA, Gully area; GD, Gully density; f_D_, Resistance coefficient of Darcy-weisbach; n_m_, Manning’s roughness coefficient; T, tailings; M, mushroom residues; S, soil; LT, loose tailings; CT, crusty tailings; TM, tailings incorporating mushroom residues; TS, tailings incorporating soil; TSM, tailings incorporating soil and mushroom residues. S.D. means the standard derivation among the repetitions (n = 3). Different letters show significant difference at *P* < 0.05 according to the LSD test for different substrates with the same rainfall event.

Mean resistance coefficient of Darcy-weisbach (f_D_) and Manning’s roughness coefficient (n_m_) varied from 0.5 to 76.5 and 0.021 to 0.253, respectively, both of which differed significantly between different substrates (p < 0.05). The value of f_D_ and n_m_ generally decreased with increased number of rainfall events. The values of f_D_ and n_m_ for S were the highest among the six substrates under the first rainfall event, followed by TM, but the f_D_ and n_m_ of TM were the greatest in all substrates under the second and the third rainfall events, which indicated the strong resistance of the underlying surface to water flow.

#### Correlations of cumulative sediment yield and cumulative runoff volume and slope micro-topography

The cumulative runoff volume, cumulative sediment yield and sediment concentration were positively correlated with relief amplitude (RA), gully area (GA) and gully density (GD), and negatively correlated with resistance coefficient of Darcy-weisbach (f_D_) and Manning’s roughness coefficient (n_m_) (Table 3). The correlation between cumulative sediment yield and RA, GA and GD were significantly higher than those between cumulative runoff volume and RA, GA and GD. Sediment concentration was strongly and positively correlated with cumulative sediment yield (correlation coefficient = 0.94), and weakly positively correlated with cumulative runoff volume (correlation coefficient = 0.37).

**Table 3:** Spearman correlation matrix between micro-topographic factors and cumulative runoff volume, cumulative sediment yield and sediment concentration.

Parameters	RA	GA	GD	f_D_	n_m_	CR	CS	SC
RA	1							
GA	0.78**	1						
GD	0.65**	0.82**	1					
f_D_	−0.71**	−0.81**	−0.72**	1				
n_m_	−0.57*	−0.76**	−0.68**	0.93**	1			
CR	0.70**	0.33	0.19	−0.33	−0.08	1		
CS	0.93**	0.90**	0.77**	−0.79**	−0.67**	0.64**	1	
SC	0.83**	0.95**	0.81**	−0.81**	−0.77**	0.37	0.94**	1

RA, Relief amplitude; GA, Gully area; GD, Gully density; f_D_, Resistance coefficient of Darcy-weisbach; n_m_, Manning’s roughness coefficient; CR, Cumulative runoff volume; CS, Cumulative sediment yield; SC, Sediment concentration. n = 18. Levels of significance: *P < *0.05*, **P < *0.01*.

## Discussion

There were two completely different sediment yield characteristics in the six substrates (Fig. 4). The sediment yield rates in LT, CT and TS were not only high, but also increased rapidly with increased rainfall duration; those of TM, TSM and S were relatively low and stable with rainfall duration. These results are in contrast with previous reports^10,43^ whereby the sediment yield rate first rapidly increased and then stabilized with rainfall duration. The differences were related to the intrinsic properties of substrates^44^. The LT, CT and TS were easily detached and transported as a result of lacking of organic matter-formed soil structure ^45^. Therefore, the rills occurred first on the middle-low parts of the three kinds of slopes, and the velocity and depth of concentrated flow in the rills were greater than the overland flow^46^, which accelerated the rills evolution. The rills of LT and CT developed in a long and deep direction resulting in RA of 38.9 mm, GA of 13.8% and GD of 12750.6 km km^−2^ after the three rainfall events. The rills in TS easily were expanded towards both sides due to the sidewall sloughs, leading to the highest GA of 28.6% and smaller GD of 3292.0 km km^−2^ almost one fourth of LT or CT. The rill morphology was completely different among LT, CT and TS, but the connectivity in rill networks was the highest relative to other substrates. Therefore, water flow velocity in the developing rills rapidly increased (Fig. 2) and accompanied by the “wave” phenomenon of sloughing from rill sidewalls or rill beds caused by gravitational erosion observed in LT, CT and TS, which drove high sediment yield rates and transported more sediment downslope than overland flows^8,47^. Young and Wiersma determined that more than 80% of the sediments eroded from hillslopes were transported in rills^48^. In this research, it was also confirmed that the sediment yields of LT, CT and TS were also the greatest.

Soil erosion is a complex interaction between rainfall and other underlying factors such as soil types, surface cover^46,49^, land use^50^, soil roughness and crusting^36,51^. For TM and TSM substrates, in the early stage of rainfall, the loose substrate particles on the surface were easy to be taken away, the non-decomposed mushroom residues physically clogged the pores surface and formed the “structural crust” or “physical crust”^52^. The permeability coefficient in TM was the lowest with only 0.019 mm min^−1^ (Table 4), similar to “partially embedded” rock fragments^22–24^ that prevented water from infiltrating and greatly increased the runoff volume (Table 1). But the overland flow was observed along the winding gap among mushroom residues on TM and TSM slopes. The flow path was extended increasing the surface roughness of n_m_ and f_D_ (Table 2), and energy was consumed decreasing the flow velocity (Fig. 2), therefore, the three-dimensional development of the rills in TM and TSM was largely limited. In particular, no obvious rills formed on TM slope except for evenly-distributed small pits induced by rainfall splash, so the GA and GD were always 0 in three rainfall events (Table 2). The slope small pits in TM and TSM were similar to the stationary rill mentioned by He *et al*.^53^, only acted as a channel for the flow, and caused less sediment yield. Reductions in sediment yield were more useful for slope stability than decreasing runoff^54^. Although a large number of studies have shown that the addition of mushroom residues as a compost to soil is beneficial because it increases soil organic matter and improves soil structure^30,31^, for example, Arthur *et al*.^31^ reported that spent mushroom compost applied to loamy sand for a period of 10 years did not signficantly reduce the water erodibility, and resulted in a notable increase (51%) in the shear strength of the topsoil. Shi *et al*.^28^ found soil incorporating a mixture of grass and wheat straw residues applied for six weeks reduced the discharge and sediment concentration, which was attributed to a higher initial aggregate stability. However, adding mushroom residues to the tailings for a short period of time in the study could not change the soil intrinsic properties, only forming “structural crust” or “physical crust” as mentioned above. TM had the maximum cumulative runoff volume of 274.5 L, but the minimum cumulative sediment yield of 4885.1 g (Table 1). This was different from previous results which indicated that high sediment yield rates were associated with high runoff rates^25,46^. The different results may be attributed to the different decomposition rates of organic materials added.

**Table 4:** Major chemical and physical properties of six substrates.

Substrate Types		LT	CT	TM	TSM	TS	S
Composition (V:V)		T	T	T:M = 2:1	T:Soil:M = 2:1:1	T:Soil = 2:1	S
Bulk Density (g/cm^3^)		1.65	1.65	1.35	1.40	1.50	1.30
Particle Size	Sand (%)	65.9	64.2	64.2	64.1	60.2	51.2
	Silt (%)	24.5	24.4	21.3	21.4	22.4	27.6
	Clay (%)	9.6	11.5	14.5	14.5	17.5	21.2
Soil Texture		Sandy loam	Sandy loam	Sand clay loam	Sand Clay loam	Sand Clay loam	Clay loam
Organic Matter (g/kg)		2.95	2.95	10.11	3.55	4.57	5.89
Permeability Coefficient (mm/min)		0.042	0.039	0.019	0.144	0.096	0.300

T, tailings; M, mushroom residues; S, soil; LT, loose tailings; CT, crusty tailings; TM, tailings incorporating mushroom residues; TS, tailings incorporating soil; TSM, tailings incorporating soil and mushroom residues.

After the three rainfall events, S had the lowest cumulative runoff volume (Table 1). This result was attributed to the differences in surface crust formation induced by rainfall. The thin seal on the surface of S was weakly resistent to raindrops penetration and the highest permeability coefficient of 0.300 mm min^−1^, which was 15.79 times higher than that of TM. Comparatively, the surface of TM was not easily penetrated because of the formation of a “physical crust” as mentioned above. Therefore, S was the least prone to generating runoff among the six substrates.

An interesting phenomenon found in this study was that the basic properties of LT and CT were similar (Table 4), but the erosion characteristics in LT were severer than CT. For example, the time to runoff initiation under the first rainfall event were 25 min and 14 min for LT and CT, respectively; but the flow velocity, runoff rate, sediment yield rate and sediment concentration in LT were all higher than those in CT, forming severe erosive slope morphology. The biggest difference between LT and CT was that LT had loose tailing particles on the surface and CT had a light natural crust from repeated sprinkling. The surface crust resulted in a reduction in unsaturated hydraulic conductivity and increase in water drop penetration time^52^. The infiltration in LT (0.042 mm min^−1^) was stronger than CT (0.039 mm min^−1^), but CT was more prone to generating runoff because of surface crust, and quickly formed rills on the lower slope under the first rainfall event. In addition, many desiccation cracks formed on the LT slope during the tailings was air-dried to field moisture content. These irregular cracks were distributed throughout the middle and upper section of the slope, and evolved into rill because of converging runoff, as observed during this experiment. The cracking along the contour line developed into trenches which intercepted runoff. Therefore, the development of rills for LT was quicker than that in CT. LT had higher RA and GA than CT, so overland flow converged at the rills thereby generating high velocity flow. This reduced the resistance of LT to water flow with lower resistance coefficient of Darcy-weisbach (f_D_) and Manning’s roughness coefficient (n_m_) than CT (Table 2). In clay soils, the formation of cracks depends on the temporal dynamics of soil water content^55^. LT and CT were both classified as sandy loam, but the swell-shrinkage of LT was severer than CT due to its initial looseness, forming complex rill network in LT. Organic matter can improve the stability of aggregates and reduce runoff and soil crust formation in silty soil^34,56^. But CT, which had the same poor organic matter content of 2.95 g kg^−1^ as LT, was consolidated only due to gravity compaction (Table 4). The resistance to erosion by CT was only slightly better than LT, but the stability of consolidation was greatly reduced in CT once it was saturated. Consequently, LT and CT had high erosion leading to high sediment yield compared to the other substrates. To our knowledge, no other studies have been conducted to investigate tailing erosion on a plot scale.

## Conclusion

This study measured the characteristics of runoff, sediment and slope erosion micromorphology of six substrates (LT, CT, TM, TSM, TS and S) related to iron tailing at a slope of 35° under three intermittent rainfall events (60, 90 and 120 mm h^−1^). With the rainfall duration, the trends in runoff rate from the six substrates were similar, but the sediment yield rates were quite different. The erosion resistance of LT, CT and TS to water erosion were the lowest. The runoff rates from LT, CT and TS were not the highest, but they had the highest sediment yield and exhibited severe erosive slope micro-topography. When the mushroom residues were added to the tailings, the resistance of tailings to water erosion was greatly improved as a result of physical obstruction or structural crusts from mushroom residues. Particularly, TM had the highest runoff volume and the lowest sediment yield, and there were hardly clear rills on its slope. S was considered to be the optimal substrate in terms of runoff volume, sediment yield and slope micro-topographic factors. However, for soilless tailings area, adding agricultural organic wastes instead of soil will be an effective way to control erosion and to improve ecological restoration.

## Materials and Methods

### Rainfall simulation system

**Figure 5:**
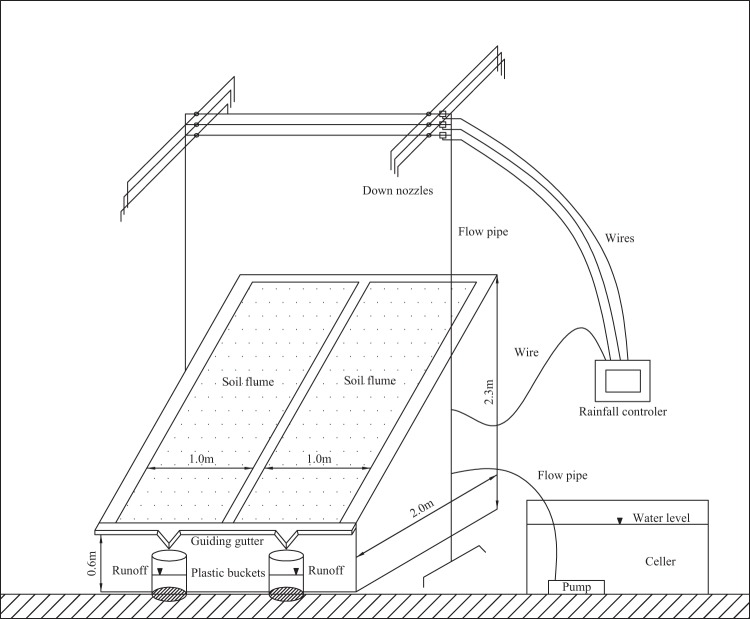
The rainfall simulator and soil flumes.

The experiment was performed in the simulated rainfall hall of National Experimental Teaching Demonstration Center, Shanxi Agricultural University, Shanxi province, China. A portable fully-automatic rainfall simulator (Model: QYJY-501) customized by Xi’an Qingyuan Measurement and Control Technology Co., Ltd. was used to simulate rainfall from 4 m high above the soil flume. The rainfall simulator has 4 sets of down nozzles, and each set includes three nozzle sizes of large, medium and small. The overlap area of 4 sets of nozzles is the effective rainfall area of 2.5 m × 2.5 m, where the raindrop distribution uniformity is > 80% and the rainfall intensity can vary from 15 to 200 mm h^−1^ by adjusting the nozzle size and water pressure. The experimental soil flume is 2.0 m long, 1 m wide, 0.6 m deep at the front end and 2.3 m deep at the rear end with a 2 cm drainage hole at the bottom. Therefore, the simulated rainfall was simultaneously carried out in the two adjacent soil flumes with 2 m × 2 m. The slope gradients ranged from 0 to 40° by adjusting the filling material height at the front and rear of soil flume. There was a groove and outlet at the front end of flume to collect the runoff and sediment from the slope. The device of the rainfall simulator and soil flumes are shown in Fig. 5.

### Experimental materials

The iron tailings used in this study were collected from a dry tailing pond at our ecological restoration base located in the south of Shanxi Province, Southeast of Loess Plateau, China. The tailings were composed of magnetic iron tailings of less than 2 mm, with a bulk density of 1.65 g cm^−3^ in the field and 2.49 g kg^−1^ of organic matter content (Table 4). The tailings were compacted as a result of poor structure, resulting in weak aeration ability, low water-holding capacity and poor scour resistance^39^. At the same time, the dry tailing pond is located in a stony mountainous area with little soil to reclaim. Edible mushroom cultivation in the area produces a lot of mushroom residues (also known as spent mushroom substrate) which is made of wood dust and corncobs with 316 g kg^−1^ organic matter, mushroom residues were selected as an amending material to improve the structure of the tailings and reduce soil and water loss. Soil was obtained from the farmland topsoil around the tailing pond, with 5.89 g kg^−1^ organic matter. This study aimed to explore soilless or less soil substrate on iron tailings slope to reduce the soil loss and promote ecological restoration.

### Experimental design

Six substrates types of loose tailings (LT), crusty tailings(CT), tailings incorporating mushroom residues (TM), tailings incorporating soil (TS), tailings incorporating soil and mushroom residues (TSM) and soil (S) were selected (Table 4). Each substrate treatment had three repetitions. The bulk density for each substrate in the soil flume was consistent with the measured value *in situ*. The soil flumes were filled with uniformly mixed materials less than 2 mm in diameter by layer. First, a layer of about 2 cm thick of sands was paved at the bottom to facilitate discharge of excess water, and a layer of cotton gauze was paved on the sands to avoid the mixing of sands and tailings. Then, the layers over cotton gauze were divided into a tailing layer of 38 cm with the bulk density of 1.65 g cm^−3^ and a substrates surface layer of 20 cm to simulate the profile structure in the field. The bulk densities of the six substrates are listed in Table 4. The filling substrates were all 35°, which was close to the slope of the tailings dumps. To ensure continuity in a vertical direction, a wire brush was used for brushing lightly between two layers to eliminate the layers effect. After finishing filling, with the exception of LT, all treatments were repeatedly sprinkled with water for one month (without runoff) to naturally settle the soil and to simulate the field natural slope surface. Therefore, before the first rainfall event, substrate surfaces formed a light natural crust except LT. In contrast with CT, the surface of LT was loose to simulate the newly abandoned tailings dump. The major properties of six substrates are shown in Table 4. The soil particle size distribution was analyzed using hydrometer method and soil texture was classified according to international soil texture taxonomy^57^, the potassium dichromate oxidation-external heating method were used to measure the soil organic matter content^43^, soil permeability coefficient was determined with constant head method^58,59^. According to the rainstorms recorded by point rainfall stations on Chinese Loess Plateau^60,61^, the erosive rainstorm of 1–2 mm min^−1^ rainfall intensity occurred generally from June to August, a field slope is usually subject to intermittent erosion during the rainy season, resulting in continuous changes in slope morphology. To simulate this, experiments under rainfall intensities of 60, 90, and 120 mm h^−1^ were conducted in a same slope chronologically^62–64^, the three rainfall intensities were named as the first rainfall event (First), the second rainfall event (Second) and the third rainfall event (Third) in sequence in this study. Before each simulated rainfall event, the same substrate was air-dried to field moisture content (8%) by considering the drying effects between rainfall events in the field. Each simulated rainfall lasted 30 min after runoff initiation.

### Experimental measurements

#### Runoff and sediment

To keep substrates in their natural state before each rainfall event, a water meter (TZS, produced by Zhejiang Top Instrument Co., Ltd. China) was used to measure the soil water content at multiple locations of slope to ensure the same initial water content of 8 ± 0.5%. The soil flumes were rained in pairs. Before a pair of soil flumes was rained on, the adjacent soil flumes on both sides were covered with plastic to prevent direct raindrop impact. When the rainfall started, the time to initial runoff was recorded for each substrate. When runoff started to flow at the outlets of the flume, the runoff and sediment samples were continuously collected with 13-L plastic buckets in 3 min intervals, and then the samples were kept for 10 hours to deposit the sediment. The runoff volumes were measured by measuring cylinders and the deposits were transferred to aluminum boxes and oven-dried at 105 °C to calculate sediment yield^43^. A total of 10 runoff and sediment samples were collected for each rainfall event. The mean sediment concentration was then calculated by the ratio of cumulative sediment yield (sum of 10 samples) to cumulative runoff volume (sum of 10 samples).

#### Flow velocity

Flow velocity was measured at 0.5 m and 1.2 m from the top of slope at the same time interval as runoff (3 min), and the two velocities were averaged. The KM_n_O_4_ tracer method^38^ was used to measure the flow velocity at the marked distances of 0.7 m. The time to tracer movement was determined with a stop-watch. A previous study indicates that the runoff velocity measured with the dyeing method is the preferential flow velocity, and the actual flow velocity of slope should be multiplied by the correction factor of 0.75^65^.

#### Slope micro-topography

The slope micro-topography was measured after each rainfall event. Slope micro-topography and rill development were monitored by marking pins and grid coordinates in combination with a high-definition digital camera (SONY, W830 made in China) and AutoCAD 2010 vectorization. Marking pins were laid out as 0.1 m × 0.1 m grid on slope before each rainfall event to ensure the pins top and the slope were exactly on the same level. After each rainfall event, a steel ruler with 1 mm accuracy was used to measure the three-dimensional coordinates of each pin to record rill development and slope micro-topography. Five indicators of Manning’s roughness coefficient (n_m_), Darcy-weisbach’s resistance coefficient (f_D_), relief amplitude (RA), gully density (GD) and gully area (GA) were used to indicate changes in slope micro-topography^66^. Calculations are presented below^66,67^.

1. Manning’s roughness coefficient and Darcy-weisbach’s resistance coefficient
  Manning’s roughness coefficient (n_m_) and Darcy-weisbach’s resistance coefficient (f_D_) reflect the resistance of the underlying surface to slope water flow. The two indicators were used to characterize the roughness of slope. The greater the resistance, the more energy required for water flow. Therefore, the energy for slope erosion and sediment transport are lower, and the sediment yield likewise becomes less^68,69^.
  Manning roughness coefficient (*n*_*m*_) and Darcy–Weisbach (*f*_*D*_) were calculated by Eqs. (1) and (2), respectively:
  1 $${n}_{m}=\frac{{R}^{2/3}{J}^{1/2}}{V}$$
  2 $${f}_{D}=\frac{8gRJ}{{V}^{2}}$$
  where *V* is mean flow velocity (m s^−1^), *R* is hydraulic radius (flow depth *h*) (m), *J* is the hydraulic gradient (sine of slope angle), and *g* is acceleration of gravity (9.8 m s^−2^).
  Flow depth (*h*) is also an important factor of surface flow. Assuming the slope flow is uniform, mean flow depth (*h*) was determined by following Eq. (3) and expressed as:
  3 $$h=\frac{q}{V}=\frac{Q}{VBt}$$
  where *h* is flow depth (m), *q* is discharge per unit width (m^3^ m^−1^ s^−1^), *t* is sampling interval time (s), *Q* is runoff volume during *t* time (m^3^), and *B* is width of water-crossing section (m).
2. Relief amplitude (RA)
  Relief amplitude reflects the ups and downs of the slope surface and is expressed with the average of all exposed heights of the steel pins after rainfall (mm). It was calculated by using Eq. (4) :
  4 $$RA=\mathop{\sum }\limits_{i=1}^{n}{H}_{i}$$
  where *H*_*i*_ is the measuring height of steel pins exposed to the substrate surface after rainfall (mm) and *n* is the numbers of steel pins set in the slope.
3. Gully area (GA) and gully density (GD)
  Gully area (GA) and gully density (GD) describes the degree of slope incision by rills. GA is represented by the area percentage of rill to slope. GD refers to the total length of erosion gullies per square kilometer, km/km^2^. AutoCAD 2010 software was used to vectorize the erosion photos to form closed graphs for slope and the rills to calculate their area and rills length. GA and GD can be calculated separately from Eqs. (5) and (6):
  5 $$GA=\frac{A}{S}\times 100 \% $$
  6 $$GD=\frac{1000L}{S}\times 100 \% $$
  where *A* is the rills area (m^2^), *S* is the slope area (m^2^), *L* is the rills length (m), and 1000 is unit conversion coefficient.

## Data analysis

The statistical analysis was performed with SPSS (version 18.0). Analysis of variance (ANOVA) was used to examine differences in the time to runoff initiation, runoff rate, sediment yield rate and flow velocity among three rainfall intensities and six substrates. Significant differences were determined using the LSD multiple range test with *p* < 0.05. A correlation matrix using spearman correlations was performed to determine the correlations between micro-topographic factors, cumulative runoff volume, cumulative sediment yield and sediment concentration^70^.
